# Assessing Search and Unsupervised Clustering Algorithms in Nested Sampling

**DOI:** 10.3390/e25020347

**Published:** 2023-02-14

**Authors:** Lune Maillard, Fabio Finocchi, Martino Trassinelli

**Affiliations:** Institut des Nanosciences de Paris, Sorbonne Université, CNRS, 75005 Paris, France; fabio.finocchi@insp.upmc.fr

**Keywords:** nested sampling, slice sampling, unsupervised clustering, harmonic potential

## Abstract

Nested sampling is an efficient method for calculating Bayesian evidence in data analysis and partition functions of potential energies. It is based on an exploration using a dynamical set of sampling points that evolves to higher values of the sampled function. When several maxima are present, this exploration can be a very difficult task. Different codes implement different strategies. Local maxima are generally treated separately, applying cluster recognition of the sampling points based on machine learning methods. We present here the development and implementation of different search and clustering methods on the nested_fit code. Slice sampling and the uniform search method are added in addition to the random walk already implemented. Three new cluster recognition methods are also developed. The efficiency of the different strategies, in terms of accuracy and number of likelihood calls, is compared considering a series of benchmark tests, including model comparison and a harmonic energy potential. Slice sampling proves to be the most stable and accurate search strategy. The different clustering methods present similar results but with very different computing time and scaling. Different choices of the stopping criterion of the algorithm, another critical issue of nested sampling, are also investigated with the harmonic energy potential.

## 1. Introduction

Function sampling can be a complicated task in multidimensional problems, especially in multimodal ones. The nested sampling (NS) algorithm [1], created in 2004 by John Skilling, allows to perform a smart sampling by reducing the multidimensional integral to a one-dimensional integral that is computationally affordable. It is mainly used in Bayesian data analysis for model selection in a variety of fields, such as cosmology [2,3] and astrophysics [4], but also in material science for potential energy exploration [5,6]. The NS algorithm consists of evolving a dynamic set of points under a hard constraint on the increasing (or decreasing) value of the explored function, which reduces the volume of the sampled space at each iteration.

One of the main difficulties of the algorithm is the choice of the new sampling points. Over the years, several methods have been used in different codes, such as uniform sampling from multi-ellipsoid [3,4], slice sampling [2,4], Galilean Monte Carlo [6], Hamiltonian Monte Carlo [6], and random walk [4,5], as well as using clustering methods, especially in multimodal problems. A more detailed overview of the various methods to find a new point can be found in Refs. [7,8]. Another challenge when using the nested sampling algorithm is choosing a criterion for convergence, i.e., deciding when the parameter space has been sufficiently explored. Evidently, the criterion for convergence depends on the problem studied. In some cases, the choice of this criterion might be quite straightforward, for example, when targeting a precise likelihood final accuracy in data analysis applications.

Nested sampling can also be employed in statistical mechanics for computing the partition function [5,6], which is a sum of the microstates of the system and depends on several macroscopic variables, such as the temperature and the volume [9]. Since one is interested not only in the partition function but also in its derivatives, which yield the thermal observables, finding a good criterion for convergence is crucial, in contrast to data analysis applications. Indeed, attaining a full convergence on the derivatives of the partition function is usually quite demanding.

In the past, the performance results of different programs implementing nested sampling were discussed [2,10,11,12]. However, the performance results of the distinct search and cluster recognition methods, and different stopping (or convergence) criteria have not been systematically compared within a single program. Here, we aim to provide a comparison of four search methods, as well as four clustering algorithms, on test cases by means of the nested_fit program, which is based on the nested sampling algorithm [1]. We will compare the accuracy and number of likelihood calls of the different methods on several benchmark tests. We will also discuss the choice of the stopping criterion for the specific case of the harmonic potential.

We briefly present the nested sampling in Section 2 and the nested_fit program in Section 3, where we also describe new search and clustering algorithms as implemented in nested_fit. We compare the different algorithms on some test examples in Section 4, including the harmonic potential. In the same section, we also compare the performance of nested_fit with other nested sampling programs written in the same programming language (Fortran). Finally, we discuss our results in Section 5.

## 2. Nested Sampling: General Concepts

### 2.1. Principle of Nested Sampling

The general need for both Bayesian model selection and partition function evaluation is the calculation of an integral *I* of a function *L* defined over *d* parameters $\theta =(\theta_{1},\ldots ,\theta_{d})$ according to a measure μ :

$$I={\int ...\int }_{\Theta }L\left(\theta \right)d\mu \left(\theta \right),$$

where Θ is the parameter space, that is θ∈Θ . Assuming that μ is a probability measure with density π , i.e., ${\int ...\int }_{\Theta }d\mu \left(\theta \right)={\int ...\int }_{\Theta }\pi \left(\theta \right)d\theta =1$ , we can write

(1) $$I={\int ...\int }_{\Theta }L\left(\theta \right)\pi \left(\theta \right)d\theta .$$

The nested sampling algorithm is based on the transformation of the multidimensional integral *I* into a one-dimensional integral:

(2) $$I=\int_{0}^{1}L\left(X\right)dX$$

via the change of variable

(3) $$X\left(L\right)={\int ...\int }_{L(\theta )>L}\pi \left(\theta \right)d\theta .$$

*X* is the normalized volume weighted by the probability π(θ) on the portion of space where the function is superior to L . L(X) is obtained by inverting the relation X(L) (Equation (3)) [8].

### 2.2. Exploration and Live Points

In practice, a quadrature via the subdivision in *M* intervals is used to approximate the integral from Equation (2). As an example, one can use the trapezium rule: we note $\Delta X_{m}=\frac{X_{m-1}-X_{m+1}}{2}$ where $0<X_{M}<\ldots <X_{2}<X_{1}<X_{0}=1$ and $X_{M+1}=0$ . In that case,

(4) $$I\approx \sum_{m}L_{m}\Delta X_{m},$$

with $L_{m}=L\left(X_{m}\right)$ . The nested sampling strategy relies on a recursive exploration of the function *L*, starting from an evolving set of *K* points—called “live points”. At each iteration, the point with the lowest value $\theta_{old}$ is discarded and replaced with a point $\theta_{new}$ that verifies $L\left(\theta_{new}\right)>L\left(\theta_{old}\right)$ . At iteration *m*, $L_{m}$ is given by $L(\theta_{old})$ .

Algorithm 1 presents the main steps of the nested sampling algorithm in the case where the trapezium rule is used to estimate integral *I*.   
**Algorithm 1:** Nested sampling with the trapezium rule.
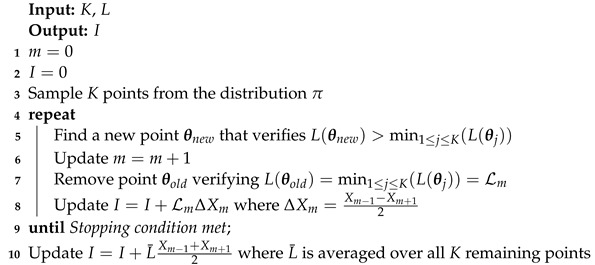


Before the first iteration, the *K* points are uniformly sampled from π(θ) . After that, at each iteration, $\theta_{new}$ is sampled from the constrained distribution $\pi^{*}\left(\theta \right)$ [8]:

(5) $$\pi^{*}\left(\theta \right)\propto \left\{\begin{array}{cc} \pi (\theta ) & \mathrm{if}L\left(\theta \right)>L\left(\theta_{old}\right),\\ 0 & \mathrm{otherwise}. \end{array}\right.$$

As a result, the volumes associated with the live points (Equation (3)) are uniformly distributed. Therefore, when removing $\theta_{old}$ , the volume shrinks by a factor t∼Beta(K,1) [1,8]—which corresponds to the distribution of the outermost value of a set of *K* samples following a Uniform([0,1]) distribution. Thus, we use the average value of the distribution of *t*, that is $\frac{K}{K+1}$ , as an estimate of $\frac{X_{i}}{X_{i-1}}$ . Therefore, at iteration *m*, the corresponding volume in the parameter space is $X_{m}\approx {\left(\frac{K}{K+1}\right)}^{m}\approx e^{-\frac{m}{K}}$ , that is, the fraction of the space that is sampled by the live points. The sequential sampled spaces defined by the constraint are nested into each other. Furthermore, at each iteration, the values of $\theta_{old}$ and $L(\theta_{old})$ are stored to be available for post processing.

We describe various methods to find $\theta_{new}$ and stopping criteria in Section 3. In some cases, unsupervised clustering algorithms are required in addition to the search methods. This is discussed in Section 3.

### 2.3. Applications

Here, we focus on two specific applications for nested sampling: (i) comparing data with a modeling function that depends on a set of unknown parameters and (ii) computing the partition function in statistical mechanics, with application to many-atom systems.

(i) In the data analysis case, the integral *I* in Equation (2) corresponds to the Bayesian evidence (also called marginal likelihood) E(M) for model M with L(θ)=P(Data|θ,M) being the likelihood and π(θ)=P(θ|M) being the prior distribution of parameters (see Equation (1)), i.e., $E\left(M\right)={\int ...\int }_{\Theta }P\left(\mathrm{Data}|\theta ,M\right)P\left(\theta |M\right)d\theta$ . The evidence can be used to compare models. Indeed, the probability of a model is calculated via the Bayes’ rule through the formula:   

(6) $$P\left(M|\mathrm{Data}\right)=\frac{E(M)P(M)}{P(\mathrm{Data})}\propto E\left(M\right)P\left(M\right),$$

where P(M) is the prior of the model [13], and P(Data) is the normalisation factor.

(ii) In statistical mechanics, we are interested in calculating the partition function or exploring energy manifolds. The classical partition function in the canonical ensemble at inverse temperature $\beta =\frac{1}{k_{b}T}$ can be expressed as

$$Z\left(\beta \right)=\int \exp\left(-\beta \tilde{E}\left(x,p\right)\right)dxdp={\left(\frac{\Lambda }{h}\right)}^{3N}\int \exp\left(-\beta E\left(x\right)\right)dx,$$

where x are the positions of the particles and p their momenta. *Z* corresponds to the integral *I* from Equation (1) with θ=(x,p) , $L\left(\theta \right)=\exp(-\beta \tilde{E}\left(x,p\right))$ and π(θ)=1 . For *N* particles in three dimensions, *Z* is therefore an integral over 6N variables. The separation in the last equality can be performed if the energy $\tilde{E}$ can be expressed as the sum of a potential depending only on the positions and a kinetic term depending only on the momenta. In that case, the latter one can be factorized out as ${\left(\Lambda /h\right)}^{3N}$ . We denote with *E* the position-dependent part of the energy $\tilde{E}$ and $Z_{x}=\int \exp\left(-\beta E\left(x\right)\right)dx$ . $Z_{x}$ can be rewritten as an integral over the energy [5,6]

$$Z_{x}\left(\beta \right)=\int \rho^{\prime }\left(E\right)\exp\left(-\beta E\right)dE,$$

where $\rho^{\prime }\left(E\right)$ is the density of states with respect to *E*, which both depend solely on the positions. In the previous equation, $Z_{x}$ is expressed as a one-dimensional integral and can therefore be calculated using nested sampling as in Equation (4):

(7) $$Z_{x}\left(\beta \right)\approx \sum_{m}w_{m}\exp\left(-\beta E_{m}\right)$$

and therefore

$$Z\left(\beta \right)\approx {\left(\frac{\Lambda }{h}\right)}^{3N}\sum_{m}w_{m}\exp\left(-\beta E_{m}\right).$$

Here, $w_{m}=\frac{1}{2}\left(\rho \left(E_{m-1}\right)-\rho \left(E_{m+1}\right)\right)$ and ρ is the cumulative density of states which corresponds to the number of states with energy lower than *E*:

$$\rho \left(E_{0}\right)=\int_{\left\{E:\;E<E_{0}\right\}}\rho^{\prime }\left(E\right)dE.$$

In Equation (7), $Z_{x}\left(\beta \right)$ , $w_{m}$ and $\exp(-\beta E_{m})$ correspond to *I*, $\Delta X_{m}$ and $L_{m}$ from Equation (4), respectively. Hence, $\rho (E_{m})$ is estimated in the same manner as $X_{m}$ , i.e., $\rho \left(E_{m}\right)\approx e^{-\frac{m}{K}}$ .

Minimizing the energy *E* is equivalent to maximizing exp(−βE) independently of the temperature [5]—indeed, exp(−βE) is a monotonic function of *E*—and we can therefore apply the nested sampling iterative procedure directly on *E*. Because the minimization process does not involve the temperature as in other methods such as Monte Carlo, simulated annealing or parallel tempering, a single run can provide the cumulative density of states ρ(E) from which the partition function can be computed at all temperatures.

From the partition function, other properties of the system can be calculated such as the internal energy $U=-\frac{\partial \log(Z)}{\partial \beta }$ and the heat capacity $C_{V}=\frac{\partial U}{\partial T}.$ In the following, we focus on the heat capacity. We take the Boltzmann constant $k_{B}$ =1 and adopt reduced units for the temperature.

## 3. The nested_fit Program and New Implementations

Nested_fit [14,15,16,17] is a program written in Fortran and originally implemented for atomic data analysis purposes that uses the nested sampling algorithm with the trapezium rule. In this section, we present various aspects of the program, such as its general structure, how to find a new point (Line 5 in Algorithm 1) or when to end a run (Line 9 in Algorithm 1), focusing on the new implementations in particular.

### 3.1. General Structure of the Program

In the input file of the program, the function to explore is specified, which can be a likelihood associated to data or an energy or a trial function, together with other parameters required for the exploration, such as the methods used for searching new points and the stopping criterion. The program returns two output files: In the first one, all the live points sampled during the run are stored. The second one contains the statistics—the value of the integral/evidence/partition function, mean and median of the parameters, number of iterations, time taken and other information.

### 3.2. Searching for New Live Points

One of the main challenges of nested sampling is finding the new point $\theta_{new}$ verifying $L\left(\theta_{new}\right)>L\left(\theta_{old}\right)$ , i.e., sampled from the distribution $\pi^{*}\left(\theta \right)$ (Equation (5)), in the multi-dimensional space with a uniform probability over the entire volume defined by the constraints. This is the task of Line 5 from Algorithm 1. Different methods have been used in different implementations of nested sampling to solve this problem, such as uniform sampling from multi-ellipsoid [3,4], slice sampling [2,4], Galilean Monte Carlo [6], Hamiltonian Monte Carlo [6] and the random walk [4,5]. In previous versions of nested_fit, a random walk called “lawn mower robot” was implemented [15].

There are a few problems that arises when using the random walk:

1. On test examples where the value of the integral is known, the random walk has difficulty finding the true value of the integral.
2. The random walk does not perform well in cases when the parameters entering L(θ) are highly correlated. In this case, the isotropic exploration of Θ leads to a drop in efficiency.
3. In the presence of multiple maxima, multiple runs are necessary to find all maxima since the random walk is generally unable to find them all in a single run.

For the last issue, a possible solution is to use unsupervised clustering recognition algorithms on the live points when the exploration starts to be inefficient [2]. Such an inefficiency comes from the analysis of the characteristics of the exploration point ensemble (mainly, the standard deviation) to determine the jump length of the random walk. When several maxima are present, the random walk often falls into regions with low values of the function. Recognizing the presence of cluster allows to choose the adapted exploration parameter for each cluster. Mean shift was therefore implemented in nested_fit [15]. The addition of this algorithm reduced the computation time by roughly an order of magnitude. A single run was enough to find all maxima (see [15] for more details). However, adjusting the mean shift parameters proved to be a complicated task. Consequently, new clustering methods were explored and implemented. They are presented in Section 3.3. To solve the first two problems, we implemented new search algorithms that are presented below.

#### 3.2.1. Uniform Search

The first search algorithm that was added to nested_fit is the uniform search around the live points. It was introduced in Ref. [18] and implemented in dynesty code [4] with cubes and spheres. The structure of this method is presented in Algorithm 2. *d* is the dimension of the problem, $L=L(\theta_{old})$ and $\theta_{n}=\theta_{new}$ . $\theta_{a}$ is the starting point which is chosen randomly for the set of *K* live points, and σ is the standard deviation of the points belonging in the same cluster as $\theta_{a}$ . If no clustering has been performed, σ is the standard deviation of all points. The method to find a new starting point in Line 13 is the same as in the random walk case [15]. *N* is the number of points rejected before a new starting point is found and $N_{N}\times N$ is the number of points rejected before a cluster analysis is performed (if clustering is used). *f*, chosen by the user, determines the size of the box proportionally to the standard deviation. Typically, *f* is chosen to be approximately 1.
**Algorithm 2:** Uniform search.
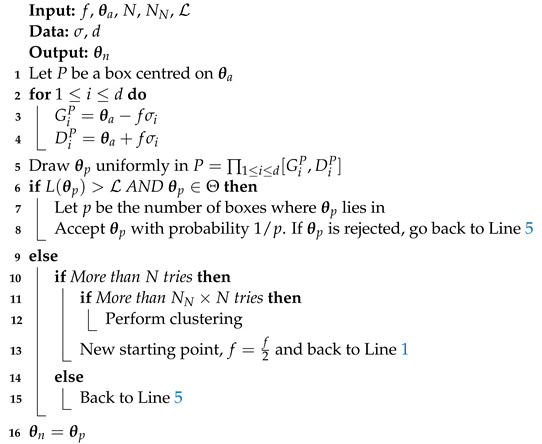


#### 3.2.2. Slice Sampling

Slice sampling [19] is used in dynesty [4] and in Polychord codes [2], among others. Slice sampling consists in uniformly choosing new exploration points on a slice of the volume defined by $L>L(\theta_{old})$ . The one-dimensional algorithm is described in Algorithm 3. The slice is initialized by randomly placing it around the starting point (Lines 1–3). In the multidimensional case, the direction is chosen after a change of coordinate to efficiently explore all the parameter space, even in the presence of a strong correlation. In practice, we use the Cholesky factorization of the covariance matrix to transform the points coordinates into new coordinates with dimensions ∼O(1) in all directions [2]. The border of the volume is found by a recursive method. Details of multidimensional slice sampling, which is adapted from the one used in Polychord [2], are described in Algorithm 4. L , $\theta_{a}$ , $\theta_{n}$ , *N* and $N_{N}$ are defined as for Algorithm 2. *d* is the dimension of the problem, and $n_{b}$ is the number of orthogonal bases used to define the slices on the live point distribution. In the following, the original space refers to Θ , while the transformed space refers to $H^{-1}\Theta =\left\{\theta^{\prime }:H\theta^{\prime }\in \Theta \right\}$ , where *H* is the Cholesky factorization of the covariance matrix. In Polychord, the multidimensional algorithm is performed in Θ with the orthonormal bases being generated in the transformed space $\left(v_{1},\ldots ,v_{d}\right)\in H^{-1}\Theta$ . Here, as shown in Algorithm 4, we transform the live points from Θ to $H^{-1}\Theta$ and carry out the slice sampling in the transformed space. The main difference between the two implementations is that, in nested_fit, the size of the slice *w* is a user-configurable parameter, while in Polychord, its value is hard coded to three times the norm of the vector $Hv_{i}$ . In both programs, the user chooses the number of steps performed—nested_fit takes a multiple of the number of dimensions, while Polychord takes the actual number of steps as a parameter. In nested_fit, if clustering is used, a cluster analysis is performed every 10∗K iterations.
**Algorithm 3:** One-dimensional slice sampling.
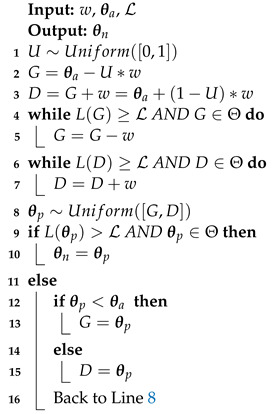

**Algorithm 4:** Multidimensional slice sampling.
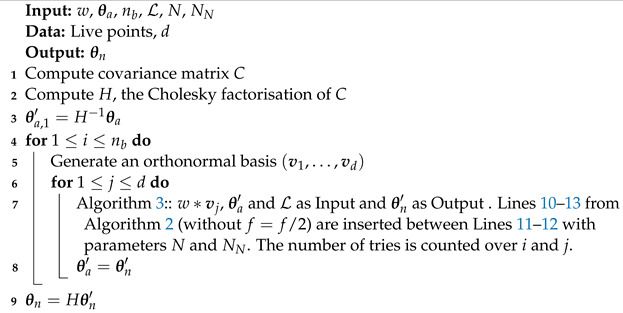


For the determination of the border of the slice, an alternative of the original slice sampling was developed and implemented in nested_fit that will be referred to as *slice sampling adapt*:

- Lines 1–3 from Algorithm 3 are replaced by Algorithm 5. The value of $m_{init}$ is defined in nested_fit.
- Lines 4–5 from Algorithm 3 are replaced by Algorithm 6. Identically, Lines 6–7 are replaced by Algorithm 6, changing *G* into *D* and the updates G=G−w and G=G+w into D=D+w and D=D−w , respectively. The value of $m_{ext}$ is defined in nested_fit.

When using slice sampling adapt, Algorithm 3 is adapted as described in Line 7 from Algorithm 4. Furthermore, we put a limit on the number of extensions that is allowed in each direction for both slice sampling and slice sampling adapt. However, this implementation of slice sampling breaks detailed balance with the potential introduction of error in the evidence evaluation. Note also that the original implementation of random walk in nested_fit partially breaks the detailed balance [15], as pointed out in Ref. [7].
**Algorithm 5:** Slice sampling adapt: initialization of the slice.
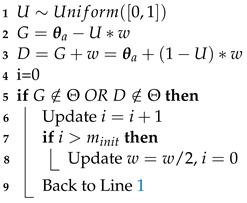

**Algorithm 6:** Slice sampling adapt: extension of the slice.
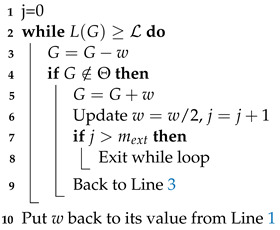


### 3.3. Unsupervised Clustering

As it was presented in the previous section, the recognition of the sampling points into different clusters can considerably improve the search algorithm. We implemented three new clustering methods in nested_fit next to the mean shift algorithm: density-based spatial clustering for applications with noise, agglomerative clustering with single linkage and k-nearest neighbors. These methods were chosen because they are unsupervised and the number of clusters is not a parameter of the method; rather, it is determined during each run of the algorithm along with the clustering. In all the following paragraphs, we use the Euclidean norm. These methods are used in Line 12 in Algorithm 2 and in Line 7 in Algorithm 4. In Algorithms 7 and 8, a min-max normalization is performed on the positions of the points at the beginning of the clustering algorithms, i.e., the coordinates are transformed according to the formula

$$\frac{x_{j}-min_{j}}{max_{j}-min_{j}}\;\forall j,$$

where $x_{j}$ is the initial *j*-th coordinate of point *x* and $min_{j}$ and $max_{j}$ are the minimum and maximum *j*-th coordinate among the points, respectively. After the transformation, all points have coordinates between 0 and 1.

#### 3.3.1. Density-Based Spatial Clustering for Applications with Noise

Density-based spatial clustering for applications with noise (DBSCAN) [20] was recently implemented as a post-processing step to recluster points sampled by MultiNest [3] since the clustering methods used in the program (X-means and k-means) were not adapted to a specific problem (microseismic events) [21]. DBSCAN requires two input parameters that are chosen by the user: a radius ϵ and a minimal number of neighbors *m*. Using these parameters, the points are divided into three categories:

- Core points. Those are the points that have at least *m* points—themselves included—within an ϵ distance.
- Border points. They are within an ϵ distance from *l* core points with 1≤l≤m−1 .
- Outliers. Those are the other points. (An outlier can be within an ϵ distance from a border point.) Outliers belong to no cluster.

The algorithm corresponding to DBSCAN is presented in Algorithm 7. When the starting point selected in the algorithm is an outlier, all of the points are used to calculate the standard deviation and covariance matrix as if no clustering was performed.
**Algorithm 7:** DBSCAN
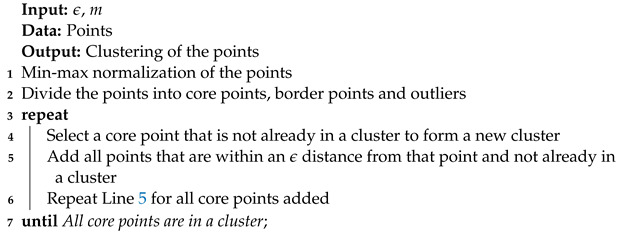


#### 3.3.2. Agglomerative Clustering with Single Linkage

Agglomerative clustering [22,23] is an iterative algorithm based on the measure of dissimilarity between two clusters. The case of single linkage is presented in Algorithm 8.
**Algorithm 8:** Agglomerative clustering with single linkage.
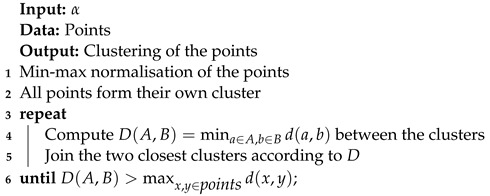


$D\left(A,B\right)=\min_{a\in A,b\in B}d\left(a,b\right)$ is the dissimilarity measure between clusters *A* and *B*. The value of α is chosen by the user.

#### 3.3.3. K-Nearest Neighbors

The k-nearest neighbors (KNN) method [24] is inspired by the method described in Polychord [2]. It is presented as Algorithm 9. The difference between our implementation and the one in Polychord is that in nested_fit, the clusters of two points are merged if there are respectively in each others’ k-nearest neighbors, while in Polychord, they are merged if one is in the other point’s k-nearest neighbors. There are no adjustable parameters for this clustering method.
**Algorithm 9:** KNN.
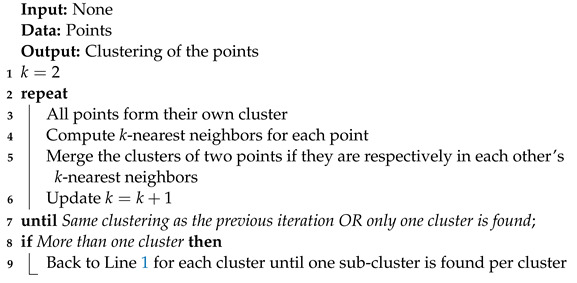


### 3.4. Stopping Criterion

In the previous version of nested_fit, only one criterion was implemented to stop the nested sampling algorithm (Line 9 of Algorithm 1): a run stopped when the ratio between the estimated total integral $I_{est,tot}$ and the calculated integral $I_{est,m}$ was below a predefined value (chosen by the user), i.e., 

(8) $$I_{est,tot}/I_{est,m}<\delta_{0},$$

with $I_{est,m}=\sum_{i=1}^{m}L_{i}\Delta X_{i}\;\mathrm{and}\;I_{est,tot}=I_{est,m}+L_{max}\left(e^{-\frac{m+1}{K}}+e^{-\frac{m-1}{K}}\right)/2\;\mathrm{at}\;\mathrm{iteration}\;m.$ The term $L_{max}\left(e^{-\frac{m+1}{K}}+e^{-\frac{m-1}{K}}\right)/2$ corresponds to an estimate by the excess of the part of the integral that is ignored if the run stops at iteration *m*.

Contrary to the case of the evaluation of the Bayesian evidence from a likelihood, this criterion is not adapted to study potential energy landscapes. Indeed, this criterion consists in evaluating the partition function at a temperature T=1 (in our units). This criterion thus does not provide a good enough convergence at lower temperatures T≪1 . To fulfill this application correctly, two additional stopping criteria were added:

1. The first criterion is equivalent to the one in Equation (8) but directly applied to the partition function. A run stops when the ratio between the estimated total partition function $Z_{est,tot}$ and the calculated partition function $Z_{est,m}$ is below a predefined value, i.e.,
(9) $$Z_{est,tot}/Z_{est,m}<\delta_{1},$$
  with $Z_{est,m}=\sum_{i=1}^{m}w_{i}e^{-\beta_{s}E_{i}}$ and $Z_{est,tot}=Z_{est,m}+e^{-\beta_{s}E_{min}}\left(e^{-\frac{m+1}{K}}+e^{-\frac{m-1}{K}}\right)/2$ at iteration *m*.
  Since the partition function depends on the temperature, a pre-defined $T_{s}=\beta_{s}^{-1}$ must then be provided by the user. Choosing the temperature equal to 1 is equivalent to using the same stopping criterion than for the evidence.
2. The second criterion is the one used in Refs. [5,6] and corresponds to stopping a run when the contribution of new points to the partition function becomes small compared to previous contributions. A run stops when the difference between the logarithm of the current contribution to the partition function $c_{m}$ and the maximum of the logarithms of previous contributions to the partition function $c_{max}$ is below a predefined value (chosen by the user), i.e.,
(10) $$c_{m}-c_{max}<\delta_{2},$$
  with $c_{m}=\log\left(w_{m}\right)-\beta_{s}E_{m}\;\mathrm{and}\;c_{max}=\max_{i\leq m}\left(c_{i}\right)\;\mathrm{at}\;\mathrm{iterationm}\;m.$ Again, this is computed at a specific temperature $T_{s}$ chosen by the user.

The main difference between the two approaches is that the stopping criterion from Equation (9) is based on the estimation of future evaluations—what is left to explore—while the stopping criterion from Equation (10) only looks at the past contributions.

## 4. Results

In this section, we compare the search algorithms on different benchmark functions, without clustering in Section 4.1, with clustering in Section 4.2, and with a brief comparison between the different clustering algorithms only (without the implementation in nested_fit) in Section 4.2.1. In Section 4.3, the different search methods are studied for model comparison applications. We then look at the evolution of the performance results with the dimension in Section 4.4. Finally, in Section 4.5, we compare the nested_fit performance results with two other programs implementing the nested sampling algorithm. Almost all calculations and analyses were done with the version 4.3 of nested_fit on a 64 bits computer with 4 CPUs of frequency 3.5 GHz, each with one single threaded core. The comparison of the computing time of the clustering methods and the comparison with other programs were conducted without parallelization. Parallelization was simply implemented via OpenMP on recursive calculation over the input parameters and the data files’ channels. On examples with real data, we observe that the 350–400% of CPU is used, while on other examples, it varies between 250% and 400%. (Since 4 CPUs are used, the maximum value is 400%).

The values of the parameter sets used for the different search methods are listed in Table 1. A thousand live points were used and eight runs were performed. The stopping criterion presented in Equation (8) was used for every function with $\ln\left(\delta_{0}\right)=10^{-4}$ for the Gaussian Shells and $\ln\left(\delta_{0}\right)=10^{-5}$ for the rest, except the harmonic potential. For the harmonic potential, the stopping criteria presented in Equations (9) and (10) were used with $\ln\left(\delta_{1}\right)=10^{-5}$ and $\delta_{2}=-10$ , respectively, with $T_{s}=0.01$ .

### 4.1. Searching New Points without Clustering Methods

In order to compare the performance results of the four search methods without the use of clustering, we consider four examples—presented in details in the following sections—for which the value of the integral *I* is known.

#### 4.1.1. Gauss

The first test function is a multivariate normal distribution with a diagonal covariance matrix with all the diagonal elements identical to each other. In *n* dimensions, it has the following form:

$$\frac{1}{{\left(2\pi \sigma^{2}\right)}^{\frac{n}{2}}}\exp\left(-\frac{1}{2\sigma^{2}}{\left(x-\mu \right)}^{T}\left(x-\mu \right)\right),$$

where $x={\left(x_{1},\ldots x_{n}\right)}^{T}$ , $\mu ={\left(\mu_{1},\ldots \mu_{n}\right)}^{T}$ is the vector of means and σ is the standard deviation. We took μ=0.5 and σ=0.01 , n=5 and a uniform prior over [0,1] for all dimensions.

#### 4.1.2. Gauss with Correlation

**Figure 1:**
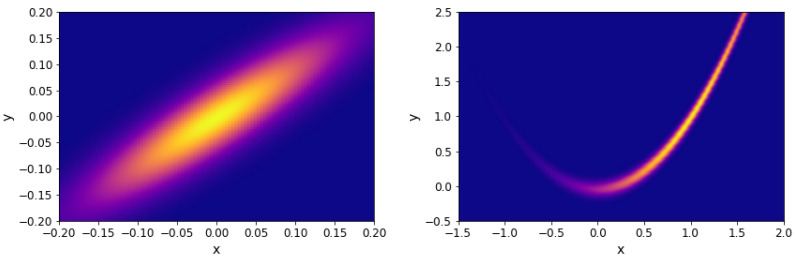
Projection of benchmark functions. **Left**: Gaussian with correlation. **Right**: Rosenbrock 2D.

The second test function is a multivariate normal distribution in *n* dimensions with a non-diagonal matrix:

$$\frac{1}{{\left(2\pi \right)}^{\frac{n}{2}}\mathrm{Det}{\left(\Sigma \right)}^{\frac{1}{2}}}\exp\left(-\frac{1}{2}{\left(x-\mu \right)}^{T}\Sigma^{-1}\left(x-\mu \right)\right),$$

where $x={\left(x_{1},\ldots x_{n}\right)}^{T}$ , $\mu ={\left(\mu_{1},\ldots \mu_{n}\right)}^{T}$ is the vector of means and Σ is the covariance matrix. We took μ=0 , n=2 ,

$$\Sigma =\left(\begin{array}{cc} 0.01 & 0.009\\ 0.009 & 0.01 \end{array}\right).$$

and a uniform prior over [−0.5,0.5] for all dimensions. The projection of the likelihood is represented in Figure 1 (left).

#### 4.1.3. Rosenbrock

The last two examples are the Rosenbrock likelihood in two and four dimensions. In *n* dimensions, the function has the following form:

$$\exp\left(-\sum_{i=1}^{n-1}\left[{\left(1-x_{i}\right)}^{2}+100{\left(x_{i+1}-x_{i}^{2}\right)}^{2}\right]\right).$$

The projection of the Rosenbrock likelihood in two dimensions is represented in Figure 1 (right). For our analysis, we considered a uniform prior over [−5,5] for all dimensions.

**Figure 2:**
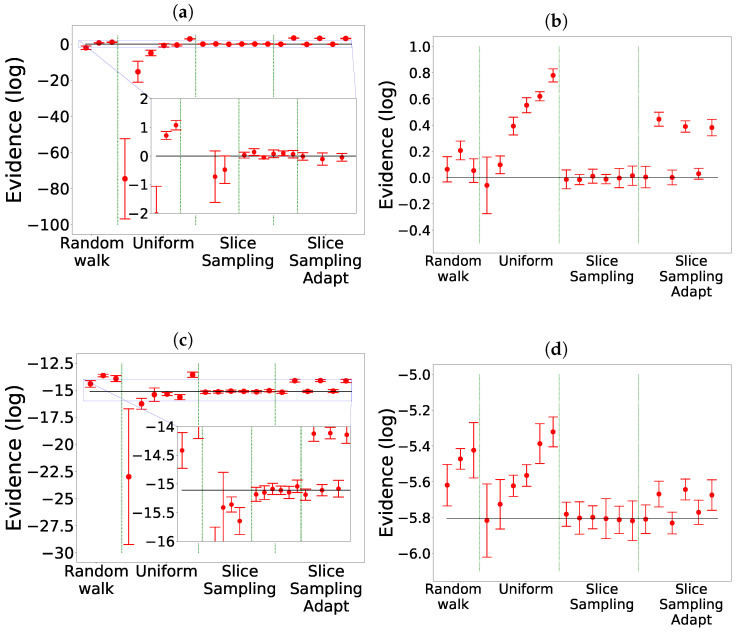
Comparison for the different search methods without clustering. The evidence is represented with the expected value represented by the black line: 0 for Gauss and Gauss with correlation, −5.804 for Rosenbrock 2D [11], −15.1091 for Rosenbrock 4D [2]. (**a**) Gauss. (**b**) Gauss with correlation. (**c**) Rosenbrock 4D. (**d**) Rosenbrock 2D.

**Figure 3:**
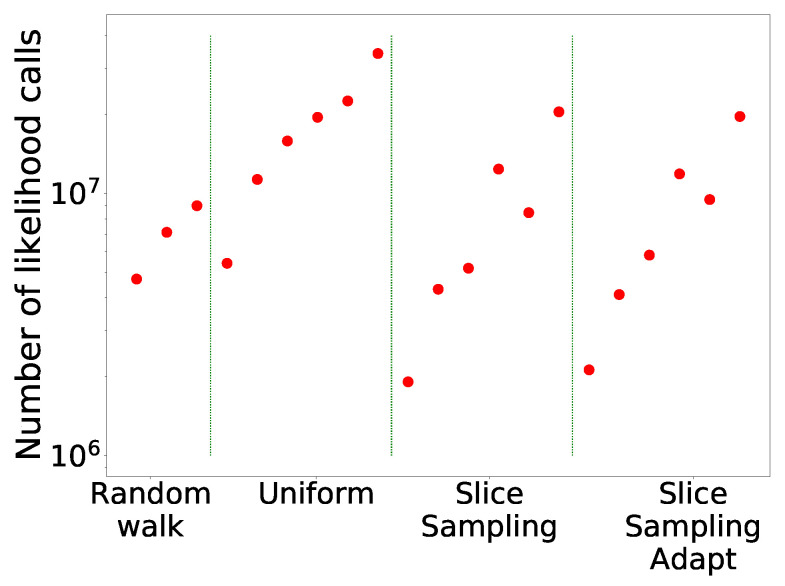
Number of likelihood calls by the different search methods for Rosenbrock 2D.

The comparison of the different search algorithms is presented in Figure 2. We can see that slice sampling performs well for all choices of the parameters and is able to recover the true value of the integral for every test function. It is also the most stable search method. For slice sampling adapt, there is a bias in the results when a too small size of the interval *w* is chosen. This bias could be a consequence of the detailed balance being broken or of the limited number of extension allowed being too small. The number of bases used seems not to have an effect on the calculated integral. When a larger interval is used, slice sampling adapt performs well. Overall, the slice sampling algorithm outperforms the random walk search. The uniform search is the least effective and robust method, which depends on the choice of the parameters in unpredictable ways. These poor performance results could be explained by the exclusion of the relevant part of the space above the likelihood constraint leading to a biased sampling of the space [18]. Moreover, as visible in Figure 3, for the uniform search, the number of likelihood calls increases with the size of the box. Furthermore, for slice sampling and slice sampling adapt, the number of likelihood calls increases with the number of bases and inversely to the size of the slice.

#### 4.1.4. Partition Function of the Harmonic Potential

For a test with a partition function, we consider the harmonic potential

$$V_{harm}\left(x,y,z\right)=\frac{1}{2}m\omega^{2}\left(x^{2}+y^{2}+z^{2}\right)$$

for a particle in three dimensions. The reason for studying this particular example is that the classical partition function can be computed analytically: $Z\left(\beta \right)={\left(\hbar \beta \omega \right)}^{-3}$ and $C_{V}=3k_{b}$ .

Therefore, we can compare straightforwardly the internal energy and heat capacity found by using nested_fit with the formulas given above. The parameters used for the different search methods are listed in Table 1. We consider a system with 7 atoms evolving in a box of side L=10au centered in 0. For such a system, the partition function is approximately $\tilde{Z}\left(V,\beta \right)\approx {\left(\hbar \beta \omega \right)}^{-3}{\left(1-\exp\left(-\frac{m\omega^{2}\beta L^{2}}{2\pi }\right)\right)}^{3/2}$ , with $V=L^{3}=10^{3}au^{3}$ . $\tilde{Z}$ is calculated for a sphere of volume *V* and depends explicitly on the volume, in contrast to *Z*. Therefore, the size of the box in which the atoms are placed is an important parameter to take into account. Indeed, there is a temperature above which the box becomes too small to correctly sample the space. More specifically, the box prevents the atoms from exploring high-energy configurations and we can therefore see a decrease in the slope of the internal energy, i.e., in the heat capacity, which we observe in our simulations.

**Figure 4:**
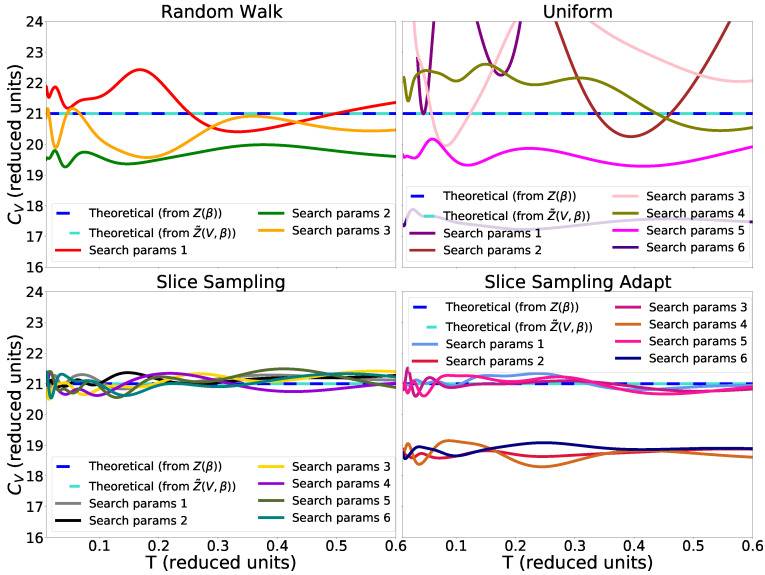
Heat capacity for the harmonic potential as computed via the distinct search methods, using a single run with 1000 live points and stopping criterion from Equation (9).

**Figure 5:**
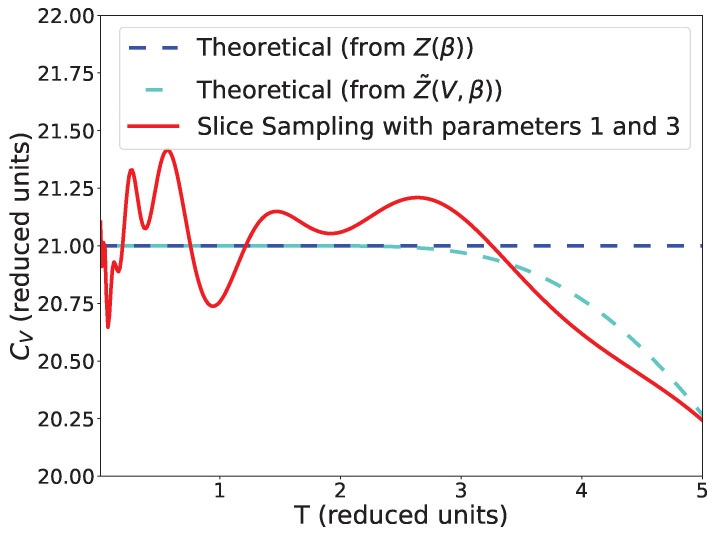
Comparison between the heat capacity for *Z*, $\tilde{Z}$ and our simulation using slice sampling with parameters 1 and 3, 1000 points and the stopping criterion from Equation (9).

In Figure 4, we see that there is no difference between the heat capacity calculated from *Z* and $\tilde{Z}$ for the temperatures we are studying. We can also see that slice sampling has good performance results as well as slice sampling adapt for the larger size of the interval *w* and with a bias with the small size of *w* (see Algorithms 4–6). Again, this bias could be a consequence of the detailed balance being broken. Finally, random walk and uniform sampling show poor performance results, and the heat capacity in the output differs significantly from one set of search parameters to another. Furthermore, while the finite volume does not have an impact on the partition function at low temperatures, at higher temperatures, *Z* and $\tilde{Z}$ no longer coincide (see Figure 5).

We tested the stopping criteria presented in Equations (9) and (10) with $\ln\left(\delta_{1}\right)=10^{-5}$ and $\delta_{2}=-10$ respectively and $T_{s}=0.01$ . When using the stopping criterion from Equation (10), we find heat capacity curves that are similar to the one from Equation (9) up to the fluctuations of the curves around the theoretical value. The number of iterations performed also vary little between the two criteria.

**Figure 6:**
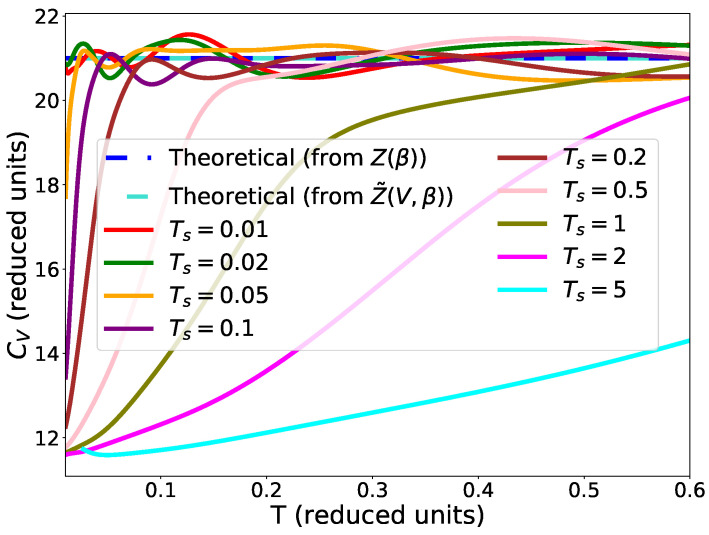
Heat capacity for the harmonic potential for different values of the temperature $T_{s}$ in the stopping criterion. 1000 live points were used. The stopping criterion from Equation (9) was used. Slice sampling with parameters 1 and 3 was used. One run was performed.

We also tested the impact of the choice of the temperature $T_{s}$ for the stopping criterion of Equation (9). The results are presented in Figure 6. We can see that the choice of the temperature $T_{s}$ has an impact on the value of the heat capacity. Indeed, we note that the correct value of the heat capacity is only found for temperatures higher than or equal to $T_{s}$ .

To summarise, slice sampling algorithms are able to correctly sample the partition function of harmonic oscillators, except when using the smaller size of the interval in slice sampling adapt. Although nested sampling can provide the partition function at all temperatures, in practice their range is limited by two parameters: the lower temperature is fixed by the precision of the convergence criterion; the higher temperature is limited by the initial size of the volume.

### 4.2. Searching New Points with Clustering Methods

To compare the performances of the search methods and clustering methods together, we consider three examples for which the value of the integral *I* is known. Before that, we compare the time taken by the different clustering algorithms alone. This comparison is performed outside the nested_fit program. The algorithms were coded in Fortran and separated from the rest of the code to perform this comparison.

#### 4.2.1. Preliminary Test on Unsupervised Clustering

**Figure 7:**
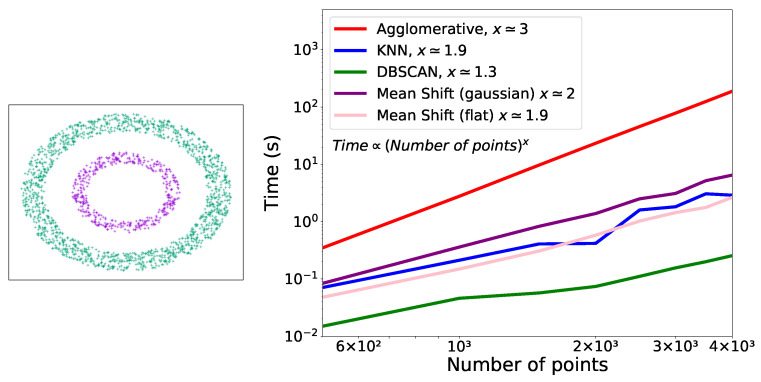
**Left**: two analysed rings. **Right**: time taken by the different clustering methods in function of the number of points used for the two rings example.

To compare the performances, in terms of execution time, of the different clustering algorithms with respect to the number of points, we used the two-ring example (represented in Figure 7 (left)). On this example, DBSCAN, Agglomerative and KNN were able to separate the two rings into two distinct clusters while Mean Shift constructed a series of clusters containing points from both rings.

Only one run was performed for each number of points. From the representation of the execution time as a function of the number of live points (Figure 7 (right)), it can be noticed that there is a cubic dependency of the time on the number of points used for Agglomerative and a quadratic or almost quadratic dependency for KNN and Mean Shift for both kernels. For DBSCAN, the dependency is between linear and quadratic.

In the next section we investigate on the performances of the search methods and clustering methods that we jointly used.

#### 4.2.2. Eggbox

The first function is the eggbox which is a synthetic and periodic function with the following form:

$$L\left(x,y\right)=\exp\left[{\left(2+\cos\left(\frac{x}{2}\right)\cos\left(\frac{y}{2}\right)\right)}^{5}\right].$$

**Figure 8:**
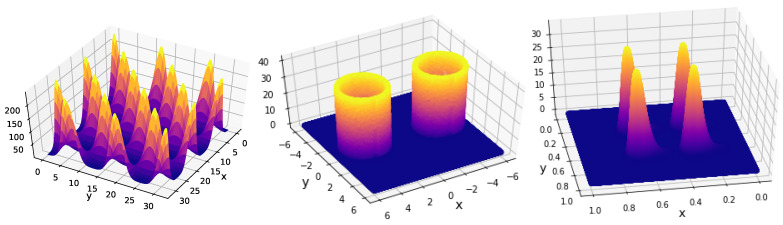
Benchmark functions. **Left**: eggbox. **Center**: Gaussian shells. **Right**: LogGamma.

This function, represented in Figure 8 (left), has several maxima and therefore, in principle, it could present a challenge if no cluster analyses are performed.

**Figure 9:**
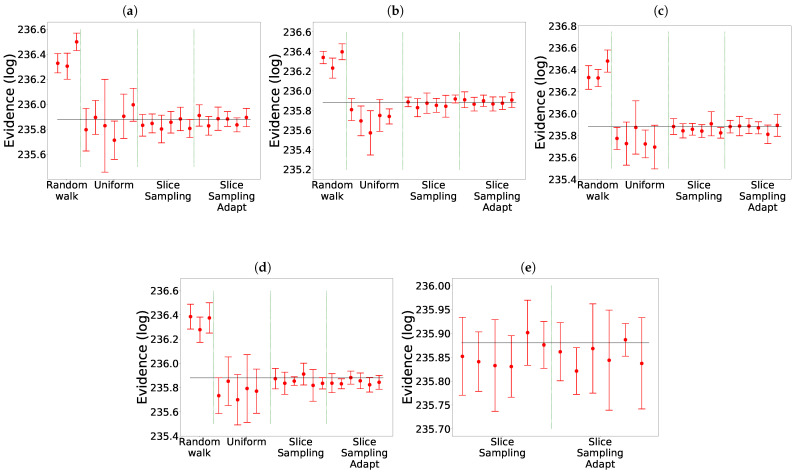
Comparison for the different search methods on the eggbox. The expected value is represented by the black line: 235.88 [3]. The missing cases failed to converge. (**a**) Mean Shift. (**b**) DBSCAN. (**c**) Agglomerative. (**d**) KNN. (**e**) Without clustering.

The results are presented in Figure 9 and Table 2, for which we took a uniform prior over [0,10π] for *x* and *y*. We notice that the choice of the clustering method does not seem to have an impact on the value found of the evidence. The main difference between the clustering methods is that the last set of parameters for the uniform only worked with Mean Shift. In this example, both slice sampling adapt and uniform are able to recover the true value of the integral most of the times. In particular, there is no bias for slice sampling adapt. Slice sampling again has good performances. The mean squared error is slightly smaller for slice sampling adapt than for slice sampling. The random walk and the uniform search have much higher mean squared errors. Detailed balanced being broken for the random walk could explain why it is unable to find the correct value for the evidence.

In Figure 9e, we can see that slice sampling and slice sampling adapt have good performances on the eggbox even if no clustering is performed. In particular, there is no bias for slice sampling adapt. When no clustering is used or when Agglomerative is used, the number of likelihod calls is slightly higher for the larger size of the slice and slightly lower for the smaller size of the slice than for the other three methods.

#### 4.2.3. Gaussian Shells

The second example we study is the Gaussian shells case. In *n* dimensions, the function is written as

$$\frac{1}{\sqrt{2\pi \sigma_{1}^{2}}}\exp\left(-\frac{(||x-\mu_{1}\left|\right|-r_{1}{)}^{2}}{2\sigma_{1}^{2}}\right)+\frac{1}{\sqrt{2\pi \sigma_{2}^{2}}}\exp\left(-\frac{(||x-\mu_{2}\left|\right|-r_{2}{)}^{2}}{2\sigma_{2}^{2}}\right),$$

where $x={\left(x_{1},\ldots x_{n}\right)}^{T}$ , $\mu_{1}=\left(-3.5,0,\ldots ,0\right)$ and $\mu_{2}=\left(3.5,0,\ldots ,0\right)$ are the centres, $r_{1}=r_{2}=2$ are the radii and $\sigma_{1}=\sigma_{2}=0.01$ the widths. The likelihood is represented in Figure 8 (center) in two dimensions. We took n=2 with a uniform prior over [−6,6] for all parameters. In this example, we had to change the value of $\ln(\delta_{0})$ in the stopping criterion (Equation (8)) from $10^{-5}$ to $10^{-4}$ otherwise convergence was never reached by the different methods.

**Figure 10:**
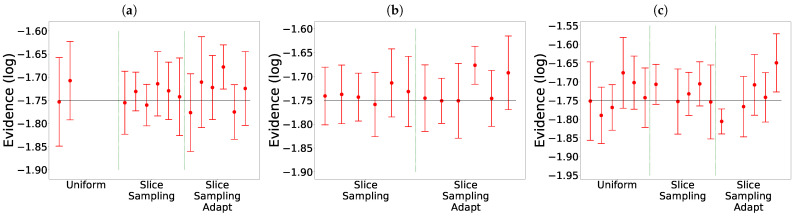
Comparison for the different search methods on the Gaussian shells. The expected value is represented by the black line: −1.75 [3]. The missing cases failed to converge. (**a**) Mean Shift. (**b**) DBSCAN. (**c**) KNN.

The results are presented in Figure 10 and Table 3. We notice that in this example the random walk search did not work whatever the clustering method. The uniform search worked well with KNN, did not work with DBSCAN and only the two smallest size of the box worked with Mean Shift. Again, slice sampling and slice sampling adapt have good performances except with KNN for the smallest size of the interval and smallest number of bases used. For this case too, the bias for slice sampling adapt is not present; the mean squared error is slightly higher for slice sampling adapt than for slice sampling and the uniform search.

#### 4.2.4. LogGamma

The third example we study is the LogGamma [18]. In *n* dimensions, the function takes the following form:

$$\frac{1}{2}\left(g_{a}\left(x_{1}\right)+g_{b}\left(x_{1}\right)\right)\times \frac{1}{2}\left(n_{c}\left(x_{2}\right)+n_{d}\left(x_{2}\right)\right)\times \prod_{i=3}^{n}d_{i}\left(x_{i}\right),$$

where

$$\left\{\begin{array}{cc} g_{a}∼\mathrm{LogGamma}\left(1,\frac{1}{3},\frac{1}{30}\right) & \\ g_{b}∼\mathrm{LogGamma}\left(1,\frac{2}{3},\frac{1}{30}\right) & \\ n_{c}∼\mathrm{Normal}\left(\frac{1}{3},\frac{1}{30}\right) & \\ n_{d}∼\mathrm{Normal}\left(\frac{2}{3},\frac{1}{30}\right) & \\ d_{i}∼\mathrm{LogGamma}\left(1,\frac{2}{3},\frac{1}{30}\right) & \mathrm{if}3\leq i\leq \frac{n+2}{2}\\ d_{i}∼\mathrm{Normal}\left(\frac{2}{3},\frac{1}{30}\right) & \mathrm{if}i>\frac{n+2}{2}. \end{array}\right.$$

We have that the probability density functions are

$$f\left(x;c,\mu ,\sigma \right)=\frac{\exp\left(c\left(\frac{x-\mu }{\sigma }\right)-\exp\left(\frac{x-\mu }{\sigma }\right)\right)}{\sigma \Gamma (c)}\;\mathrm{and}\;f\left(x;\mu ,\sigma \right)=\frac{1}{\sqrt{2\pi }\sigma }\exp\left(-\frac{{\left(x-\mu \right)}^{2}}{2\sigma^{2}}\right)$$

for the LogGamma(c,μ,σ) and the Normal(μ,σ) respectively, where Γ is the gamma function. We took n=2 and n=10 with a uniform prior over [0,1] for all parameters. The likelihood is represented in Figure 8 (right) in two dimensions. This function is asymmetric and heavy-tailed with four maxima [18].

**Figure 11:**
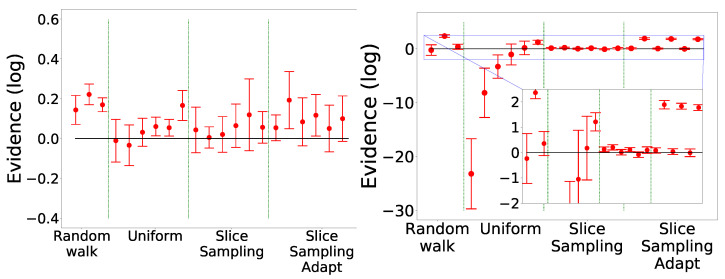
Comparison for the different search methods on the LogGamma 2D (**left**) and 10D (**right**). The expected value is represented by the black line: 0 [18]. Mean Shift was used as the clustering algorithm.

The results are presented in Figure 11. We notice that again slice sampling is able to recover the true value of the evidence in most cases. Moreover, we again see the bias for slice sampling adapt, especially in the 10D case. The random walk is unable to recover the true value of the integral in two dimensions. In ten dimensions, it manages to find the correct value in two cases. Lastly, the uniform search still presents a wide variation of the results with the size of the box, more visible in the 10D case. In ten dimensions, even though clustering was activated, no analysis was performed for the uniform search except for the last set of parameters. Furthermore, slice sampling and slice sampling adapt encountered difficulties finding new points at the beginning leading to the algorithm stopping early. Often, multiple tries were necessary to obtain full convergence on the eight runs.

### 4.3. Bayesian Model Comparison for Data Analysis

**Figure 12:**
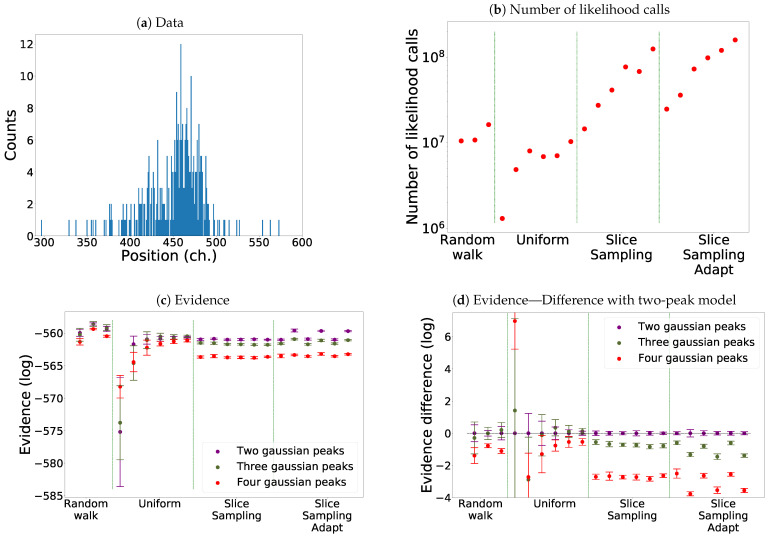
Comparison for the different search methods for model comparison. One thousand live points were used. Eight runs were performed each time. Mean Shift was used as the clustering algorithm. (**a**) The data used for the model comparison. (**b**) The number of likelihood calls by the different methods for the model with four peaks. (**c**) Comparison of the evidence of the three models using the different search methods. (**d**) Difference between the evidence of the three models with the two-peak model.

Here, we study the example of a likelihood associated to real data which corresponds to a high-resolution X-ray spectrum of a helium-like intrashell transition of uranium [25]. They are represented in Figure 12a. The data set is characterised by low statistics with the presence of many local maxima of the likelihood function.

For our model, we take *n* Gaussian peaks with the same width and a flat background. There are 2n+2 parameters: the position and amplitude of each peak, the width (common to all peaks) and the value of the background. n! maxima of the likelihood are present corresponding to the permutations of the position of the *n* peaks.

We choose n=2,3,4 . The evidence for one peak is small compared to the other cases; therefore we are not considering the one-peak model here.

In Figure 12b, we see that slice sampling and slice sampling adapt make more likelihood calls than the other two methods. We also notice that the number of calls increases proportionally to the number of bases used and inversely to the size of the interval. Slice sampling adapt requires slightly more calls than slice sampling.

As visible in Figure 12c,d and Table 4, we again notice that slice sampling is the most stable method and uniform sampling the least stable one. Indeed, we see that slice sampling always chooses the model with two peaks with probabilities around 60–65%. Slice sampling adapt also favours the two-peak model; however, the probabilities of the different models vary with the size of the slice. The uniform search tends to slightly favour the three-peak’ model except for the three smallest sizes of the box where in one case, it chooses the model with four peaks and the model with two peaks in the other two. The random walk gives similar probabilities to the model with two peaks and the one with three peaks. Finally, we notice that for the uniform search and the random walk, the uncertainties of the evidence of the different models overlap while this is not the case for both versions of the slice sampling algorithm. To note, a difference of 0.9 in log corresponds to a *p*-value of 0.05 in favour of the more complex model [26].

### 4.4. Scaling with the Dimension

To study the evolution of the performance with the dimension of the problem, we consider the Gaussian case presented in Section 4.1.1 in two, four, eight, sixteen and thirty-two dimensions. We consider four cases: slice sampling with 5 bases and w=1 , the uniform search with a box size of f=0.4 , the random walk with 160 steps of size 0.1 and the random walk with the number of steps corresponding to five times the dimension of the problem and a step size of 0.1 . The case in thirty-two dimensions is the same for both random walk.

**Figure 13:**
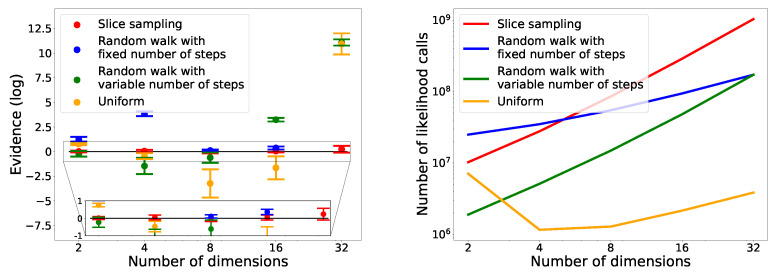
Comparison of evolution of different search methods with dimension on the Gaussian (Section 4.1.1) in two, four, eight, sixteen and thirty-two dimensions. Eight runs were performed for each case. On the **left**, the computed values are represented. The expected value is represented by the black line: 0. On the **right**, the correspondent number of likelihood calls are reported.

In Figure 13, we see that only slice sampling was able to recover the true value of the integral in every case. For the other methods, they were not able to recover the correct value, except the random walk with a variable number of steps in two dimensions. We also notice that for slice sampling and both random walk, the number of likelihood calls increases with the number of dimensions. In the case of the uniform search, the number of likelihood calls decreases between two and four dimensions before increasing with the dimension from four to thirty-two dimensions. This initial decrease could be due to the box being too big in two dimensions, leading to the rejection of many points. In the case of random walk, for a fixed number of steps, the number of calls increases slower than for a variable number of steps.

### 4.5. Comparison with Polychord and MultiNest

Finally, we compare the performance results of nested_fit with the ones of two other programs, implementing the nested sampling algorithm and based on the same programming language (Fortran): Polychord [2,27] and MultiNest [3,28,29].

The comparison is conducted on two examples: the eggbox presented in Section 4.2.2 in two dimensions, and the Gaussian presented in Section 4.1.1 in two, four, eight, sixteen and thirty-two dimensions. For nested_fit, we chose to use slice sampling with w=1 and 5 bases, that is, the number of steps performed is five times the number of dimensions. The stopping criterion is the one from Equation (8) with $\log\left(\delta_{0}\right)=10^{-5}$ . For Polychord, the number of steps was set to five times the number of dimensions. For MultiNest, we did not use the importance nested sampling option. For both Polychord and Multinest, we set the tolerance to $10^{-5}$ , which is equivalent to the stopping criterion in nested_fit. (In Polychord and Multinest, the stopping criterion used is $\left(I_{est,tot}-I_{est,m}\right)/I_{est,m}<tol$ where tol is the tolerance. In Polychord, the remaining part of the evidence is estimated with the mean value of the likelihood while it is estimated with the maximum value of the likelihood in nested_fit.) We used 1000 live points in all programs. Clustering was used for the eggbox (KNN in the case of nested_fit, the default one for Polychord and Multinest). Eight runs were performed for each program and example to evaluate the evidence uncertainty. Polychord and Multinest were used in pure-Fortran mode. For all three programs, the number of iterations performed were roughly the same.

In Table 5, we see that nested_fit is able to recover the true value of the integral while Polychord and Multinest slightly overestimate it. The values of the uncertainties—corresponding to the standard deviation of the eight runs—vary little between the programs. Moreover, MultiNest requires much fewer likelihood calls than the two other programs, and Polychord requires slightly fewer likelihood calls than nested_fit.

**Figure 14:**
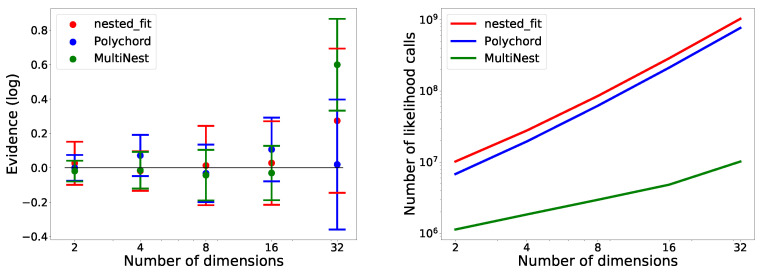
Comparison of nested_fit, Polychord and MultiNest on the Gaussian (Section 4.1.1) in two, four, eight, sixteen and thirty-two dimensions. Eight runs were performed for each case. On the **left**, the computed values are represented. The expected value is represented by the black line: 0. On the **right**, the correspondent number of likelihood calls are reported.

For the Gaussian functions, in Figure 14 (left), we see that, while Polychord and nested_fit are able to recover the value of the integral for all dimensions, MultiNest fails in 32 dimensions. In Figure 14 (right), we notice that, again, nested_fit requires slightly more likelihood calls than Polychord—around 1.5 times as many. MultiNest is the program needing the fewest likelihood calls; however, we observe a bias in the values found.

The errors presented in Figure 14 (left) and Table 5 were estimated from eight runs. However, Polychord and Multinest estimate this error with each run. We find that the two methods lead to errors of the same order of magnitude.

## 5. Discussion

After the development and implementation of new search and clustering methods, we test and compare their use with the nested sampling algorithm. For the clustering method, we saw that a crucial factor to consider when choosing which method to use is the computation time, especially for a large number of sampling points *K*. Indeed, there is a wide disparity in the scaling of the different methods with *K*, while they have comparable final performance results on cluster recognition efficiency. As we can see in Figure 7, for a large number of points, it is preferable to use DBSCAN since it is the fastest method. Agglomerative should not be used in that case, as the time taken to perform the clustering can outweigh the benefit of performing said clustering. In any case, from the study of the different cases, it emerges that the method used must be chosen in accordance with the problem studied.

About the search methods, from the test of a variety of very different benchmarks, it results that slice sampling is the most robust and accurate method to find new live points; it manages to find the expected value most of the time. Uniform search has very unstable results and can vary considerably between two sets of parameters. The random walk is seldom able to find the correct value. These observations could be explained by the fact that slice sampling effectively explores the whole space by extending the slice, while the random walk only explores the space within around a standard deviation of its initial point. Similarly, in the uniform search, where the new point is randomly chosen in a box centered on a random live point, large portions of the space may be ignored. For slice sampling adapt, a bias is present for some choices of the parameters. This bias, appearing for the smaller size of the interval, could be due to the volume not being fully explored due to the slice being too small. This bias and the poor performances of the random walk could also be a consequence of the detailed balance being broken. We also saw that, while performing cluster analyses is needed in some cases with the uniform search and the random walk, slice sampling and slice sampling adapt are able to work with and without clustering except in the LogGamma case. Furthermore, we saw that slice sampling still has good performance when increasing the dimension.

In terms of the run time of the different algorithms, we can see that slice sampling adapt requires a higher number of likelihood calls than slice sampling for the same set of parameters. This is probably due to the fact that, in slice sampling, when the border of the slice is outside the bound, the extension is stopped without calculating the likelihood, while in slice sampling adapt, the likelihood is calculated for every extension inside the bound (see Algorithms 3 and 5). We also saw that, for those two methods, the number of calls increases with the number of bases used—which is expected since augmenting the number of bases is equivalent to augmenting the number of steps performed. The size of the slice also has an impact on the number of likelihood calls. Indeed, there are more calls when smaller intervals are chosen. A possible explanation is that, for a smaller interval, the slice needs to be extended more times. For the uniform search, we noticed that the number of calls tends to increase with the size of the box. This can be due to the fact that when the box size is increased, there is also a potential increase in the proportion of its volume lying below the likelihood constraint, which decreases the probability of sampling a new point verifying the nested sampling constraint. There is also a potential increase in the number of points inside the box leading to an increase in the probability of rejecting the new point. For the random walk, the number of likelihood calls increases with the number of steps. The size of the step does not have much of an impact on the number of calls.

When choosing the search method, one must make a compromise between the accuracy of the method and the number of likelihood calls, especially for the computationally expensive likelihood. Indeed, for slice sampling, which gives the most reliable and stable results throughout the examples we studied, its number of calls is very dependent on the dimension of the problem. Other factors must be taken into account when choosing a method, such as its ability to reach convergence, as we saw that for the LogGamma example, slice sampling, while providing the best results, had difficulties finding new points at the start. One has also to carefully consider the parameters of the method, as we saw that choosing a smaller size of the interval for slice sampling adapt can result in a bias, or increasing the number of bases in slice sampling considerably increases the number of likelihood calls.

In the case of model comparison for a given data set, as we can see in Table 4, the different search methods do not favor the same model. For this specific case, we do not know the value of the evidence or the correct model. Evaluating which method performs best is thus more complex that on test examples. In light of the results on the test cases, the use of slice sampling should be favored in this case too.

For the harmonic potential, we saw that slice sampling is the best method to calculate the partition function and its derivatives. We also saw that restricting the space to a finite volume, which is mandatory with nested_fit, means that the partition function cannot be fully explored above a certain temperature that depends on the volume considered.

Concerning the choice of the stopping criterion and more specifically of the virtual temperature $T_{s}$ when computing a partition function, we saw that it must be smaller than the physical temperature. It would be interesting to see if this observation still holds when studying more complex systems, especially one that presents phase transitions. The stopping criteria from Equations (9) and (10) give similar results, and both can be used to study an energy. However, the stopping criterion from Equation (8)—which is equivalent to choosing the criterion from Equation (9) with a temperature of 1—should not be used.

Finally, when different codes are compared, we saw that, while MultiNest is faster than Polychord and nested_fit, it is not able to find the correct value for the Gaussian in thirty-two dimensions. It may be preferable to use MultiNest when the number of dimensions is not too high, while Polychord or nested_fit perform better in higher dimensions. This corresponds to the conclusion found in Ref. [2] when comparing Polychord and MultiNest. We also notice that Polychord requires fewer likelihood calls than nested_fit and might therefore be preferable. For both programs, the number of likelihood calls is of the same order of magnitude.

## Figures and Tables

**Table 1:** Parameter sets for the different search methods used for the comparison.

**Random walk**	Set 1	Set 2	Set 3			
Search parameter 1 (Size of the step)	0.1	0.2	0.1			
Search parameter 2 (Number of steps)	20	20	40			
**Uniform**	Set 1	Set 2	Set 3	Set 4	Set 5	Set 6
Search parameter 1 (Size of the box *f*)	0.1	0.2	0.3	0.4	0.5	1
Search parameter 2 (Number of steps)	1	1	1	1	1	1
**Slice sampling**	Set 1	Set 2	Set 3	Set 4	Set 5	Set 6
Search parameter 1 (Size of the interval *w*)	1	0.2	1	0.2	1	0.2
Search parameter 2 (Number of bases)	1	1	3	3	5	5
**Slice sampling adapt**	Set 1	Set 2	Set 3	Set 4	Set 5	Set 6
Search parameter 1 (Size of the interval *w*)	1	0.2	1	0.2	1	0.2
Search parameter 2 (Number of bases)	1	1	3	3	5	5

**Table 2:** Comparison of the mean squared error for the different search and clustering methods on the eggbox. The expected value for the evidence is 235.88 [3].

	Mean Shift	DBSCAN	Agglomerative	KNN	No Clustering
Random walk	0.2546	0.2009	0.2530	0.2195	/
Uniform	0.0087	0.0342	0.0189	0.0149	/
Slice sampling	0.0026	0.0010	0.0012	0.0016	0.0012
Slice sampling adapt	0.0010	0.0004	0.0009	0.0016	0.0012

**Table 3:** Comparison of the mean squared error for the different search and clustering methods on the Gaussian shells. The expected value for the evidence is −1.75 [3].

	Mean Shift	DBSCAN	KNN
Uniform	0.0002	/	0.0016
Slice sampling	0.0007	0.0003	0.0008
Slice sampling adapt	0.0015	0.0015	0.0031

**Table 4:** Probabilities in % of each model (two, three and four Gaussian peaks) for the data in Figure 12a calculated with the evidences shown in Figure 12c. Equation (6) was used with equal prior for each model. The search methods are presented in the same order as they appear in Table 1.

	Random Walk				Uniform								
Two peaks	50.1	40.8	39.1		0.1	89.2	46.4	34.8	37.0	36.7			
Three peaks	37.4	40.4	48.0		0.4	5.0	40.9	49.0	41.5	41.8			
Four peaks	12.5	18.8	12.9		99.5	5.9	12.7	16.2	21.5	21.5			
	**Slice Sampling**							**Slice Sampling Adapt**					
Two peaks	60.9	63.3	64.4	64.7	67.1	65.4		61.2	77.6	65.9	79.3	61.6	78.2
Three peaks	35.0	32.3	31.4	31.1	29.0	30.0		33.8	20.6	29.4	18.4	33.6	19.5
Four peaks	4.0	4.4	4.2	4.2	4.0	4.7		5.0	1.8	4.7	2.3	4.8	2.2

**Table 5:** Comparison of nested_fit, Polychord and MultiNest on the eggbox function. Expected value for the evidence is 235.88 [3].

	Evidence	Number of Likelihood Calls
nested_fit	235.879±0.117	10,030,647
Polychord	236.071±0.043	6,520,085
MultiNest	235.950±0.049	456,459

## Data Availability

Raw data can be provided on demand.
