# Correction: Maillard et al. Assessing Search and Unsupervised Clustering Algorithms in Nested Sampling. *Entropy* 2023, *25*, 347

**DOI:** 10.3390/e26010055

**Published:** 2024-01-09

**Authors:** Lune Maillard, Fabio Finocchi, Martino Trassinelli

**Affiliations:** Institut des Nanosciences de Paris, Sorbonne Université, CNRS, 75005 Paris, France

There was an error in the original publication. The authors noticed an error in the expression of $\tilde{Z}$ in the paper [1].

A correction has been made to Section 4, Section 4.1.4, second paragraph:

$$\tilde{Z}\left(V,\beta \right)\approx {\left(\hbar \beta \omega \right)}^{-3}{\left(1-\exp\left(-\frac{m\omega^{2}\beta L^{2}}{2\pi }\right)\right)}^{3/2}.$$

In the original publication, there was a mistake in Figure 5. The above error in the expression of $\tilde{Z}$ affects the curve of Figure 5. Corrected Figure 5 appears below.

There were two errors in the original publication. There are two sentences in Section 4.1.4 and Section 5, respectively, that do not apply to the new curve in Figure 5.

A correction has been made to Section 4, Section 4.1.4, second paragraph:

The sentence “We also see that this decrease appears at a lower temperature for the theoretical limit of the partition function $\tilde{Z}\left(V,\beta \right)$ with explicit dependence in the volume than for our simulations.” should also be removed.

A correction has been made to Section 5, sixth paragraph:

The sentences “As visible in Figure 5, there is a difference between the theoretical heat capacity calculated from $\tilde{Z}$ and our simulation. This may be due to the fact that the space is a box for the simulation and a sphere for $\tilde{Z}$.” should also be removed.

The authors state that the scientific conclusions are unaffected. This correction was approved by the Academic Editor. The original publication has also been updated.

## Figures and Tables

**Figure 5:**
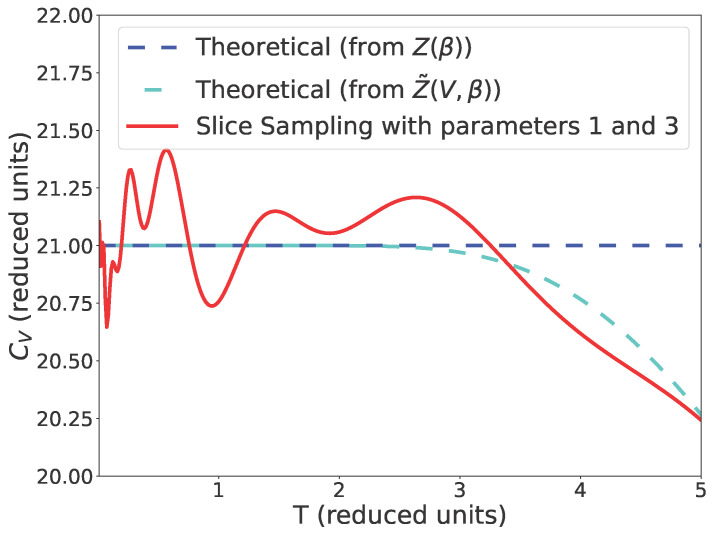
Comparison between the heat capacity for *Z*, $\tilde{Z}$ and our simulation using slice sampling with parameters 1 and 3, 1000 points and the stopping criterion from Equation (9).
